# Chronic, Progressive Back Pain, Fever and a Noticeable Paravertebral Mass

**DOI:** 10.4269/ajtmh.2011.10-0672

**Published:** 2011-03-04

**Authors:** Homarh Villaverde, Eduardo Gotuzzo, Carlos Seas

**Affiliations:** Instituto de Medicina Tropical Alexander von Humboldt, Universidad Peruana Cayetano Heredia, Lima, Peru; Departamento de Enfermedades Infecciosas, Tropicales y Dermatológicas, Hospital Nacional Cayetano Heredia, Lima, Peru

A 46-year-old woman presented with an 8-month history of fever, lumbar pain, and a slowly growing right paravertebral mass. She reported regular consumption of unpasteurized goat cheese. The physical examination revealed a fluctuant right paravertebral mass and marked pain on palpation over the lumbar spine at the L3-L4 level. A computed tomography (CT) scan of the spine revealed erosions of the end plates of L3 and L4 and the antero-superior angle of L3, narrowing of the intervertebral space between L3 and L4, and new bone formation (Figure 1). Widening of the right iliopsoas muscle was also observed on magnetic resonance imaging (MRI) of the spine (Figure 2). A tube agglutination test for brucellosis was positive at 1/640, and a CT-guided percutaneous drainage of the paravertebral mass yielded purulent material from which *Brucella melitensis* was isolated in culture. Doxycycline, gentamycin, and rifampicin were started with good clinical response. Bacterial vertebral osteomyelitis may be caused by pyogenic bacteria, *Mycobacterium tuberculosis*, and *B. melitensis*. Tuberculosis predominantly affects the thoracic spine; paravertebral masses are common, but new bone formation is unusual. Pyogenic vertebral osteomyelitis, mainly caused by *Staphylococcus aureus*, presents very similarly to brucellosis both clinically and radiographically. Brucellosis caused by *B. melitensis* is acquired mostly by eating contaminated dairy products. Vertebral osteomyelitis and iliopsoas abscess are recognized complications of untreated brucellosis.^1^ Triple antibiotic therapy seems to improve clinical outcome.^2^

**Figure 1.**
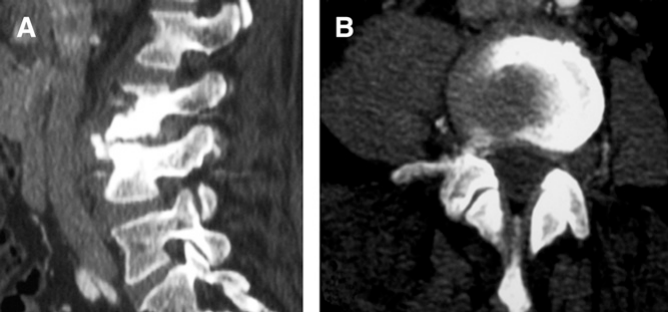
(**A**) CT scan of the spine showing erosions on the antero-superior angle of the vertebral body of L3, narrowing of the intervertebral disc space between L3 and L4, erosions on the end plates of these two vertebral bodies, and new bone formation. (**B**) Widening of the right iliopsoas muscle.

**Figure 2.**
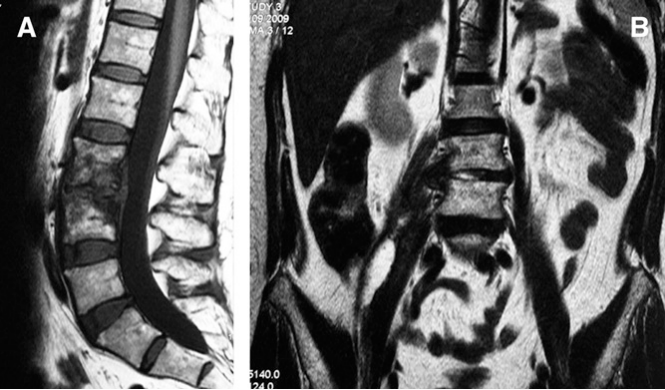
(**A**) MRI of the spine. Hypointensity on T1-weighted images at the L3-L4 level and narrowing of the L3-L4 intervertebral disc space. (**B**) Hyperintesity on T2-weighted images and marked widening of the right iliopsoas muscle.

## References

[1] Colmenero J Ruiz-Mesa J Plata A Bermúdez P Martín-Rico P Queipo-Ortuño M Reguera J 2008 Clinical findings, therapeutic approach, and outcome of brucellar vertebral osteomyelitis Clin Infect Dis 46 426 433 18181740 10.1086/525266

[2] Bayindir Y Sonmez E Aladag A Buyukberber N 2003 Comparison of five antimicrobial regimens for the treatment of brucellar spondylitis: a prospective, randomized study J Chemother 15 466 471 14598939 10.1179/joc.2003.15.5.466

